# Searching for COVID-19 Antibodies in Czech Children—A Needle in the Haystack

**DOI:** 10.3389/fped.2020.597736

**Published:** 2020-11-12

**Authors:** Marketa Bloomfield, Iva Pospisilova, Tamara Cabelova, Anna Sediva, Marketa Ibrahimova, Klara Borecka, Martin Magner

**Affiliations:** ^1^Department of Pediatrics, First Faculty of Medicine, Charles University and Thomayer's Hospital, Prague, Czechia; ^2^Department of Immunology, Second Faculty of Medicine, Charles University and Motol University Hospital, Prague, Czechia; ^3^Department of Clinical Chemistry, Thomayer's Hospital, Prague, Czechia; ^4^Laboratory of Immunology, Thomayer's Hospital, Prague, Czechia; ^5^Department of Pediatrics and Inherited Metabolic Disorders, First Faculty of Medicine, Charles University and General University Hospital, Prague, Czechia

**Keywords:** SARS-CoV-2, COVID-19, antibodies, seroprevalence, pediatric, children

## Abstract

During the COVID-19 pandemics of 2020, caused by the severe acute respiratory syndrome coronavirus 2 (SARS-CoV-2), both adults and children were shown to mount a specific antibody response to the virus. As infected children often exhibit mild symptoms or even remain asymptomatic, they are likely to be under tested for the direct presence of the virus. Mapping the SARS-CoV-2 antibodies frequency informs more accurately on the disease prevalence and helps guide the protective and therapeutic strategies. To date, only few seroprevalence studies included children. In the Czech Republic, in April 2020, the overall SARS-CoV-2 seroprevalence was estimated not to exceed 1.3%. In July and August, 2020, we screened 200 children (0–18 years of age), who attended the pediatric department of a large hospital in Prague for various COVID-19-unrelated reasons, for the presence of SARS-CoV-2 antibodies. Zero seropositive subjects were found. Therefore, we hereby report a low (<0.5%) seroprevalence amongst children in Prague, as of August, 2020.

## Introduction

In the Czech Republic, the first 3 cases of COVID-19 disease caused by the SARS-CoV-2 virus were identified on March 1st, 2020. As of July 1st, 2020, the total of 12,000 cases (diagnosed from nasal swabs by real-time polymerase chain reaction, RT-PCR), including ~1,000 children, were confirmed amongst the national population of 10.5 million, resulting in the cumulative incidence of 114 cases/100,000 inhabitants and over 350 deaths (1). Children account for ~20% of the Czech population, yet represent only about 8% of all cases. While the country incidence rate was on the lower end compared to other European countries (2), no more than 6% of the population had been tested for the presence of the virus by RT-PCR to the same date (1).

Given the possible asymptomatic course of the disease (3–5), together with symptom-based testing and variable performance of the RT-PCR tests, the actual disease burden may be underestimated and better quantified by serologic studies. Although the majority of children experience a less severe course of COVID-19 disease (4, 6), they have been shown to be prolific virus shedders, both via respiratory and oro-fecal routes (7, 8). As such, they represent an important source of community transmissions. Both adults and children have been shown to produce virus-neutralizing antibodies in response to the infection (7, 9). In fact, SARS-CoV-2 IgG antibodies are detected in 90–99% of infected people 2 weeks after symptoms onset (10). Assuming that the presence of anti-viral antibodies indicates the existence of at least some level of post-infectious immunity, an accurate estimate of childhood seroprevalence may aid the modeling of epidemiologic predictions and guide the appropriate anti-epidemic measures, including stay-at-home orders and schools shutdowns. On the other hand, recent data point toward a rapid decay of virus-specific antibodies in the sera of patients after acute viral antigenic exposure, particularly in asymptomatic or mild cases (11–13). However, the immune protection from SARS-CoV-2 infection is not limited to antibody-mediated mechanisms. For example, the virus-specific CD4^+^, CD8^+^ T memory cells, as well as mucosal IgA likely represent other elements of at least partial post-infection protection (14).

Several seroprevalence surveys of various extend and target cohorts have been conducted globally (3, 5, 15–17), however children remain largely underrepresented. A Czech population-based, predominantly adult seroepidemiological survey carried out in April, 2020, utilizing the point-of-care antibody detection kits, estimated the maximum overall seropositivity as low as 1.3% (18). Here, we set out to survey anti-SARS-CoV-2 antibody frequency amongst children and adolescents residing in Prague, Czech Republic, using commercially available immunoassays.

## Patients and Methods

The study is a cross-sectional single-time point serology survey. Two hundred children were recruited from patients aged 0–18 years, who were examined between July 3rd and August 19th, 2020, in the Department of Pediatrics in Thomayer's hospital in Prague, Czech Republic, for various general pediatric reasons. All the children who were undergoing blood sampling regardless of this study and only if their guardians/themselves gave their consent were included. The exclusion criteria were residency outside of the Czech Republic and known primary antibody deficiency. Thomayer's hospital serves the area of Eastern Prague and neighboring parts of Central Bohemian region with the population of ~400,000 residents, including some 80,000 children. The cohort was analyzed for demographic parameters, i.e., age, sex, current medical condition (acute infectious illnesses, acute non-infectious conditions, or ongoing care/chronic illness), known previous contacts with COVID-19 RT-PCR positive person(s), previous positive RT-PCR test, and the history of possible COVID-19 symptoms (e.g., fever, respiratory illness, nausea, vomiting, diarrhea, tiredness, headaches, joints and muscle pain, loss of taste and smell). The exclusion criteria were residency outside of the Czech Republic and known primary antibody deficiency.

The Elecsys Anti-SARS-CoV-2 assay from Roche Diagnostics (19) was selected as the prevalence-screening test. Venous blood (2–5 ml) was drawn into tubes containing a clot activator and serum gel separator and analyzed within 2 h. The assay is a fast fully-automated ECLIA (electrochemiluminescence immunoassay) developed for Cobas e601 analyzer. It uses one-step sandwich method allowing the binding of high affinity mature antibodies independently of their subclasses. The target antigen is recombinant nucleocapsid protein of SARS-CoV-2. The measurement of anti-SARS-CoV-2 antibodies was performed according to the manufacturer's instructions, and the results were reported as numeric values in the form of cutoff index (COI = signal sample/cutoff). The specificity of this test is >99.8%, sensitivity of 99.5% (14 days post-PCR confirmation) according to the manufacturer, and it displays no cross-reactivity to other human coronaviruses. In case of a positive result of the screening test (COI ≥1.0), a confirmation by another subclass-dependent assay was intended. In order to measure the SARS-CoV-2 IgA and IgG antibodies, we selected ELISA (enzyme linked immunosorbent assay) from Euroimmun (20, 21). The assay uses indirect double-steps sandwich format with fixed antigen, which is a recombinant protein of the S1 domain (spike protein) of SARS-CoV-2. An enzyme-labeled anti-human IgA or IgG conjugate is applied to detect the bound antibodies. The results were reported as numeric values in the form of a ratio (the optical density of the sample/internal calibrator). A ratio ≥1.1 is considered positive (borderline from 0.8 and 1.09). Anti-SARS-CoV-2 IgA ELISA has a specificity of 92% and sensitivity of 98.6% (10 days post-PCR confirmation); anti-SARS-CoV-2 IgG ELISA has a specificity of 99.6% and sensitivity of 94.4% (10 days post-PCR confirmation) according to the manufacturer. There are no cross-reactions with other human coronaviruses. Both the Elecsys Anti-SARS-CoV-2 assay and Euroimmun Anti-SARS-CoV-2 ELISA performances have been independently evaluated (22). We used both assays for the detection of anti-SARS-CoV-2 antibodies in four SARS-CoV-2 RT-PCR positive COVID-19 patients at different time points from symptom onset.

## Results

**Figure 1:**
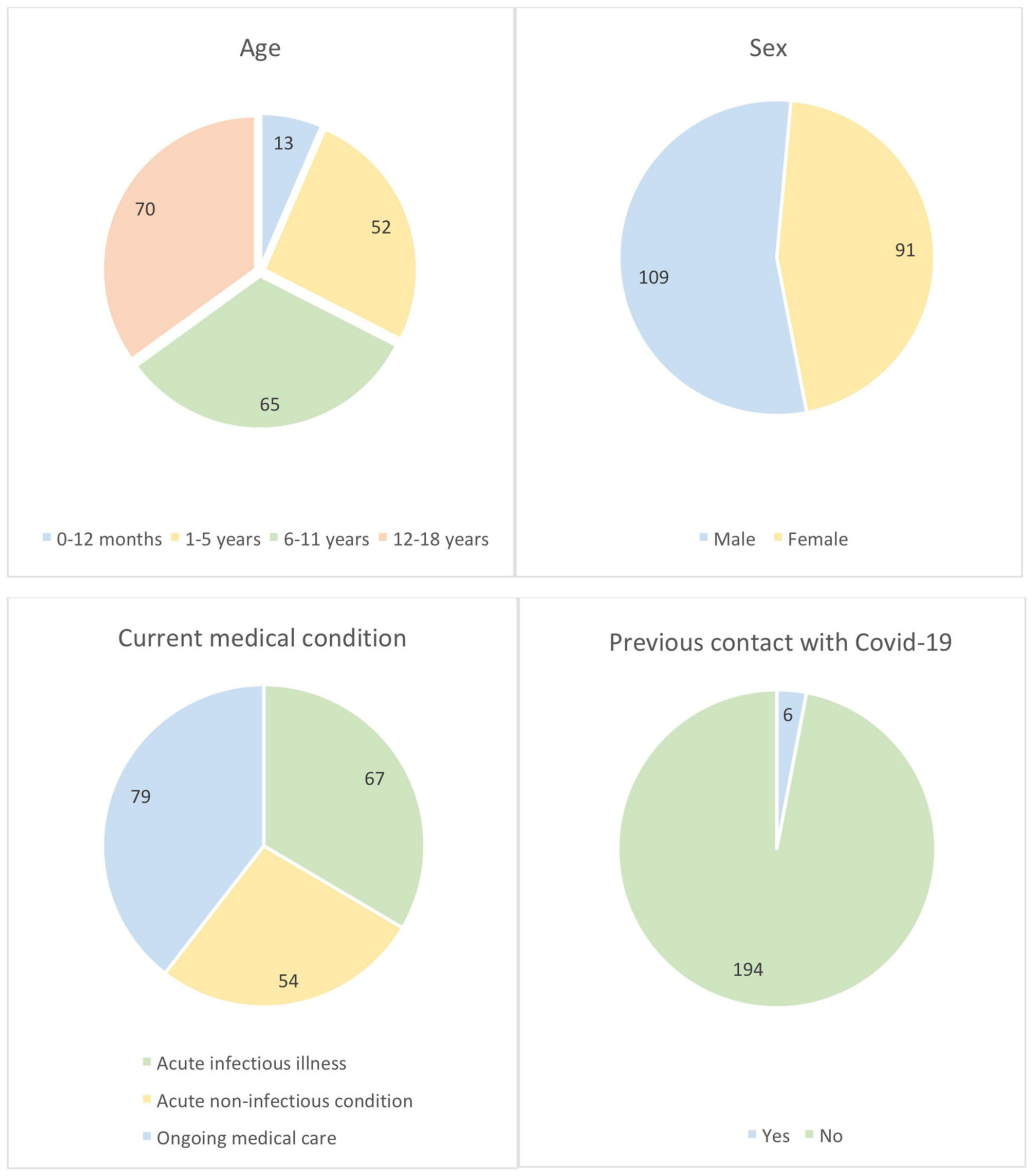
The characteristics of the cohort (*n* = 200).

The cohort age stratification was 0–12 months (13/200; 6.5%), 1–5 years (52/200; 26%), 6–11 years (65/200; 32.5%), and 12–18 years (70/200; 35%) with male/female ratio 54.5/45.5%. 6/200 (3%) subjects reported previous known contact with COVID-19 RT-PCR positive person. No subject had previously received RT-PCR nasal swab test. Sixty-seven (33.5%), 54 (27%), and 79 (39.5%) subjects were tested during the course of acute infectious illness (e.g., viral or bacterial infections), acute non-infectious conditions (e.g., injury, pre-surgery testing, collapse, allergic reactions, bronchial asthma exacerbation), and as part of ongoing medical care (e.g., failure to thrive, endocrinopathy, chronic gastrointestinal, respiratory, nephrologic, hematologic, neurologic diseases), respectively (Figure 1). Eighteen (9%) subjects were receiving immunosuppressive therapy at the time of the testing (azathioprine, methotrexate, anti-TNFα, corticosteroids). Approximately 30% of patients reported having experienced possible COVID-19-related ailments since January 2020, however the precise analysis of this data was hindered by incomplete ascertainment due to a large proportion of guardians or patients reporting unspecific or uncertain symptoms.

All the study subjects tested negative with ECLIA immunoassay. Randomly selected samples from the cohort were re-tested with ELISA immunoassay and were consistently found to be negative across the study period.

**Figure 2:**
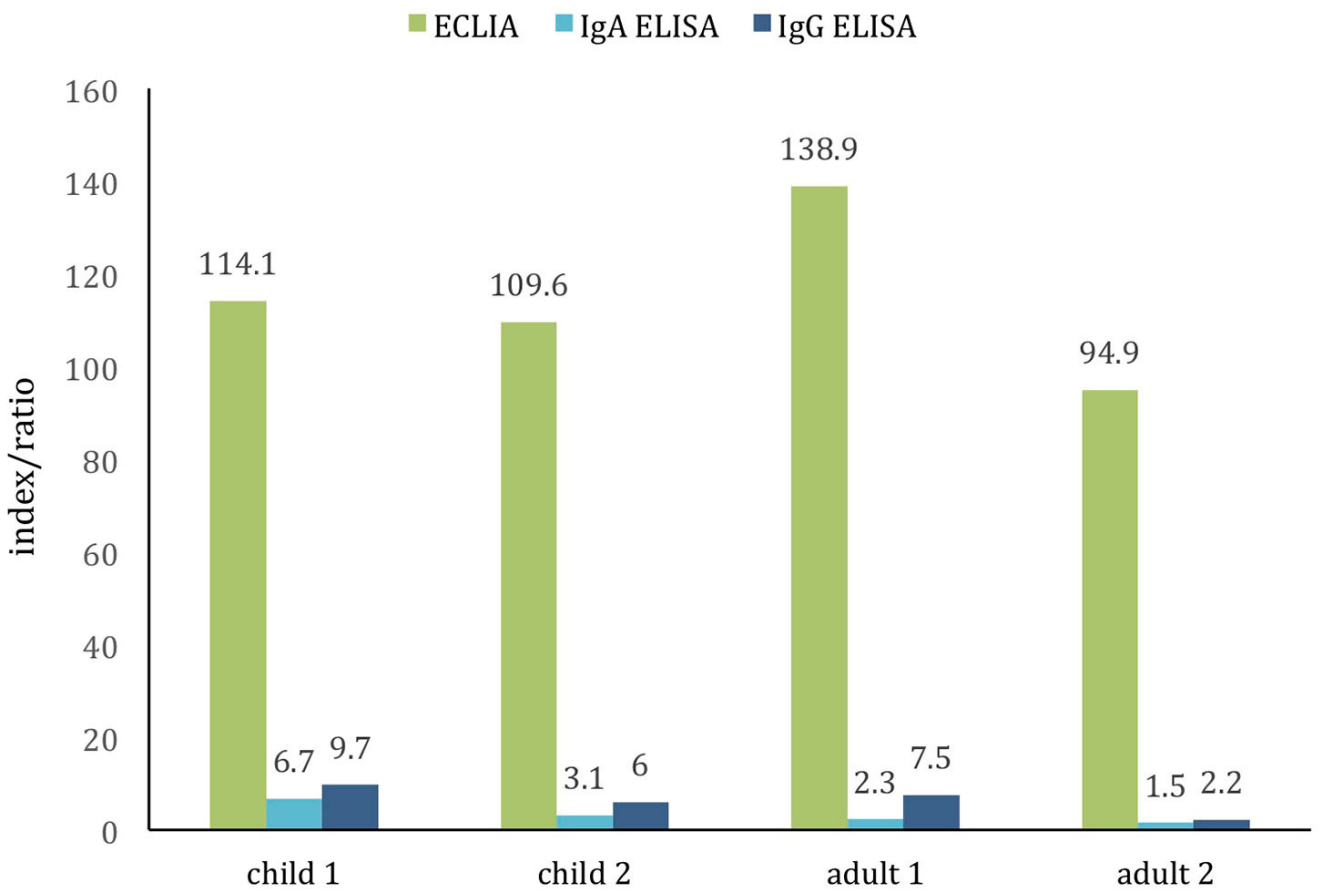
Comparison of two commercial assays for detection of SARS-CoV-2 antibodies in the positive control cohort of PCR-confirmed COVID-19 children (age 4 and 8 years) and adults (age 37 and 50 years). The cutoff value of both tests is ~1.0. All samples are positive (samples collected ≈2–8 weeks from symptoms onset).

Given the absence of positive results, samples from patients with the history of PCR confirmed and clinically manifested COVID-19 were examined as positive controls. Sera from two children, aged 4 and 8 years, and 2 adults, aged 37 and 50 years, collected 2–8 weeks from symptom onsets tested positive with both ECLIA and ELISA assay (Figure 2) indicating the tests' unskewed reliability.

Thus, when adjusted for the assay performance (99.5% sensitivity), the seroprevalence of SARS-CoV-2 in the studied population would range between 0 and 0.5%.

## Discussion

The seroprevalence studies during epidemics provide invaluable data maintaining surveillance over the disease activity. Although some of the over 100 published serosurveys on COVID-19 included children (1, 15, 16, 23, 24), the pediatric population was often disproportionately underrepresented. Moreover, the studies differ in quality, are often burdened by various levels of non-random sampling bias and vary in sensitivity/specificity of the used testing method. Such heterogeneity is illustratively reflected in the overall seroprevalence estimates ranging from 0.4 to 59.3% (25). This renders the assessment of the actual seroprevalence in children largely prediction-based, relying on suboptimal data.

The only larger scale pediatric-only survey to date reported the prevalence of only ≈1% amongst ~1,000 children in Seattle (26). In our cohort of 200 children, not a single case of seropositivity was found. The SARS-CoV-2 seroprevalence in the Czech Republic was estimated not to exceed 1.3% overall, and 3.3% in the most affected regions (1) in April, 2020. Accounting for the cumulative incidence rates, this corresponds well with reports from other European regions, which estimated a similar seroprevalence of ~0.8–1.4% per every 100 confirmed COVID-19 cases per 100,000 inhabitants (i.e., overall seroprevalence of 3% in France, 4.6% in Spain, 6% in Belgium, 10.8% in Geneva) (5, 23, 24, 27). Deriving from this data, we report a lower than expected frequency of seropositive children, likely not exceeding 0.5%. Due to the lack of positive subjects in our cohort, a larger population sample would be necessary to provide a methodologically rigid pediatric population prevalence estimate.

Nevertheless, our results align with previous observations, that seroprevalence may be lower in children compared to adults (5, 23, 24). Illustratively, the largest population-based SARS-CoV-2 seroprevalence study from Spain, one of the most severely affected European country, reported the overall 4.6% seroprevalence, and 3.8% seroprevalence in population 0–19 years of age in May, 2020 (5). Because very high post-infection seroconversion rates have been consistently documented across the publish studies in both adults and children (over 90%) (7, 10), it is unlikely that the lower pediatric seroprevalence would be due to lesser ability to elicit the antibody response. On the contrary, children might be more efficient producers of neutralizing antibodies compared to adults (7, 28). The incidence rates/age disparity may be linked to lower childhood susceptibility to the virus (29), possibly due to immune cross-protection from other coronaviruses frequently acquired by children (30), or to the lower expression of angiotensin-converting enzyme 2 receptor in nasal epithelia, which is used by SARS-CoV-2 as cellular entry point (31). Moreover, individuals with asymptomatic or mildly symptomatic course of the infection (typical for children) were shown to loose the circulating anti-SARS-CoV-2 antibodies rapidly, even to resume seronegativity as early as 2–3 months into the convalescence (11, 13). Therefore, the absence of circulating virus-specific antibodies may not be an accurate indicator of past infection. Of note, at least six children from our cohort reported a previous contact with SARS-CoV-2 RT-PCR positive, clinically symptomatic person (more than 2 weeks prior to antibody testing), yet developed neither symptoms nor were the SARS-CoV-2 antibodies detected in their sera.

Our study included a proportion of children receiving immunosuppressive treatment. While the antibody responses may be dampened, compared to healthy individuals, it has been shown that the humoral responses to vaccines, including influenza, are not abolished in these patients (32). Furthermore, patients receiving anti-TNFα were shown to be capable of induction of autoantibodies, such as the formation of anti-nuclear antibodies, double-strand DNA, and others, advocating the retained ability to produce antigen-specific antibodies (33). Therefore, these children were not excluded from the study.

We acknowledge that the study cohort is limited in size and that its representativeness is selectively biased by recruiting the subjects from patients either receiving ongoing medical care or seeking acute care. Chronically ill patients may adopt more stringent behavioral precautions in order to limit infection exposure compared to their healthy peers. Also, at the time of heightened public awareness of infectious risk the majority of non-essential visits to the hospital were discouraged, introducing further bias to the study group. Therefore, the results from this cohort must be interpreted with caution, particularly when extrapolating to the general pediatric population.

In summary, we demonstrate a low SARS-CoV-2 seroprevalence in a cohort of children visiting a hospital in Prague during 5th and 6th month from the beginning of COVID-19 epidemics in the Czech Republic. Although not unbiased, our work suggests that the majority of the children in Prague remained seronegative, as of August 2020, which may have important epidemiologic implications, particularly in the imminent wake of the second peak of the epidemics, as well as for the prospective vaccine employment.

## Data Availability Statement

The raw data supporting the conclusions of this article will be made available by the authors, without undue reservation.

## Ethics Statement

The studies involving human participants were reviewed and approved by Ethical committee of Thomayer's Hospital, Prague, Czech Republic. Written informed consent to participate in this study was provided by the participants' legal guardian/next of kin.

## Author Contributions

MB designed the study, supervised the sample collection, analyzed the data, and wrote the manuscript. IP performed the ECLIA assay and co-analyzed the data. TC organized the sample collection and co-analyzed the data. MI performed the ELISA assay and co-wrote the manuscript. AS, KB, and MM co-organized the study and reviewed the manuscript. All authors contributed to the article and approved the submitted version.

## Conflict of Interest

The authors declare that the research was conducted in the absence of any commercial or financial relationships that could be construed as a potential conflict of interest.
